# Advanced Nuclear Energy Pathways for a Net‐Zero World: Fuel Cycles, Reactors, and Policy Readiness

**DOI:** 10.1002/gch2.70110

**Published:** 2026-04-29

**Authors:** Reda A. Haggam, Ravikumar Jayabal, Sekar S, Manikandan Ayyar, Manzoore Elahi M. Soudagar, Santhamoorthy M, Saravanan Rajendran, Shanmugapriya D, Rajkumar Sivanraju

**Affiliations:** ^1^ Department of Chemistry Faculty of Science Islamic University of Madinah Madinah 42351 Saudi Arabia; ^2^ Department of Mechanical Engineering Academy of Maritime Education and Training AMET University Chennai Tamil Nadu India; ^3^ Department of Mechanical Engineering Rajalakshmi Engineering College Chennai Tamil Nadu India; ^4^ Department of Chemistry Centre for Material Chemistry Karpagam Academy of Higher Education Coimbatore Tamil Nadu India; ^5^ Centre for Promotion of Research Graphic Era (Deemed to be University) Dehradun Uttarakhand India; ^6^ Department of Mechanical Engineering and University Centre for Research & Development Chandigarh University Mohali Punjab 140413 India; ^7^ School of Chemical Engineering Yeungnam University Gyeongsan Republic of Korea; ^8^ International Center of Nanotechnology and Functional Materials Instituto de Alta Investigación, Universidad de Tarapacá Arica 100000 Chile; ^9^ Vicerrectoría de Investigación y Postgrado Universidad de La Serena La Serena Chile; ^10^ Department of Mechanical Engineering, Faculty of Manufacturing Institute of Technology Hawassa University Ethiopia

**Keywords:** alternative fuels, advanced reactors, proliferation resistance, sustainability, waste management

## Abstract

This review examines the contributions of alternative nuclear fuels and advanced reactor technologies to enhancing the sustainability, safety, and resource efficiency of nuclear energy systems during the transition to a low‐carbon future. A thorough synthesis of the existing literature was undertaken, with an emphasis on Thorium‐based fuels, uranium‐233, minor actinides, and innovative reactor configurations encompassing molten salt reactors (MSRs), small modular reactors (SMRs), and fast breeder reactors (FBRs). The investigation integrates various dimensions, including technical performance metrics, fuel cycle attributes, environmental ramifications, and pertinent global policy frameworks. MSRs exhibit thermal efficiencies exceeding 45% while incorporating online reprocessing capabilities, whereas SMRs provide advantages in modular deployment and intrinsic safety features. FBRs facilitate closed fuel cycles and the transmutation of long‐lived isotopes. Lifecycle emissions consistently remain below 12–20 gCO_2_e/kWh. Empirical case studies from India, China, and the United States substantiate this field's technical readiness and regulatory progress. Advanced fuel compositions and reactor systems offer a plausible trajectory toward sustainable nuclear energy. The achievement of this potential is contingent upon the evolution of contemporary regulatory frameworks, active public engagement, and adequate financial mechanisms. Integrating technology, policy, and public trust is essential to making nuclear energy a key pillar of global decarbonization.

## Graphical abstract

Integrating alternative nuclear fuels with advanced reactor technologies enables more sustainable energy systems by reducing waste, improving safety, and strengthening proliferation resistance.

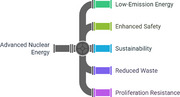

## 1 Introduction

Nuclear energy is recognized as a preeminent technology for providing extensive, low‐carbon electricity, serving an essential function in the global shift towards sustainable energy paradigms [1]. Considering escalating global energy requirements, exacerbating climate change apprehensions, and the urgent necessity to curtail greenhouse gas emissions, nuclear energy presents a reliable and uninterrupted power source that can effectively supplement variable renewable energy resources such as wind and solar [2]. The imperative to decarbonize the energy sector has rekindled scholarly and public interest in nuclear energy, particularly given its capacity to deliver dispatchable baseload power devoid of the carbon emissions linked to fossil fuel consumption [3].

Notwithstanding these merits, the long‐term viability of nuclear power is predicated upon the successful navigation of several formidable challenges. The dependence on uranium‐235 as the principal fissile material imposes constraints due to the limited availability of natural reserves and the associated complexities of its extraction and enrichment processes [4]. Furthermore, producing high‐level radioactive waste engenders substantial environmental and safety dilemmas, necessitating the implementation of secure and sustainable geological disposal methodologies [5]. The public perception of nuclear energy remains ambivalent, particularly after catastrophic incidents such as Chornobyl and Fukushima, which have exacerbated concerns regarding reactor safety, radiation risks, and preparedness for potential disasters [6]. An additional complication arises from the spectre of nuclear proliferation. Conventional nuclear fuel cycles, especially those involving plutonium, embody inherent risks related to diversifying materials for military applications [7]. Consequently, enhancing nuclear technologies' proliferation resistance is paramount for sustaining international security and ensuring the judicious deployment of nuclear energy resources [8].

Intensified research and development initiatives have emerged within alternative nuclear fuels and innovative reactor designs in response to these intricate challenges. These advancements aspire to augment fuel efficiency, diminish waste generation, enhance safety protocols, and mitigate proliferation risks, bolstering the overall sustainability of nuclear energy systems [9]. Alternative fuels, such as Thorium and uranium‐233, exhibit promising attributes, including greater natural abundance, reduced longevity of waste, and intrinsic resistance to proliferation. When integrated with advanced reactor technologies, these fuels have the potential to enhance the efficacy and safety of nuclear systems markedly [10].

Next‐generation reactor designs, encompassing MSRs, SMRs, and FBRs, represent a profound advancement from traditional light‐water reactors (LWRs). For instance, MSRs function at low pressure and employ liquid fuel salts, facilitating inherent safety features and permitting on‐site fuel reprocessing [11]. SMRs provide a modular and scalable approach that can abbreviate construction durations and expenditures while improving safety through passive cooling mechanisms [12]. In contrast, FBRS optimize the utilization of uranium resources by generating more fissile material than they consume and can transmute long‐lived waste into isotopes with shorter half‐lives [13].

The amalgamation of these technological innovations has the potential to fundamentally revolutionize the nuclear energy paradigm. By effectively closing the nuclear fuel cycle, reducing waste generation, and enabling flexible deployment strategies, these advancements pave the way towards a more resilient and ecologically responsible atomic future [14]. However, the extensive implementation of these systems is contingent upon resolving economic and regulatory impediments [15]. Substantial initial capital requirements, protracted development timelines, and intricate licensing processes may dissuade investment and impede deployment. Consequently, comprehensive policy frameworks and international collaboration are imperative to stimulate innovation, guarantee safety, and cultivate public confidence in nuclear energy technologies [16].

Moreover, the ecological impact of nuclear energy necessitates rigorous evaluation. Although emissions during operation are minimal, optimizing the entire lifecycle encompassing extraction and fuel production, reactor functionality, and decommissioning processes is imperative to guarantee overall sustainability improvements [11]. The management of nuclear waste continues to pose a significant challenge, accompanied by ongoing investigations into partitioning and transmutation methodologies that can potentially diminish the volume and hazardousness of the waste generated [17].

Recent research has improved the understanding of new types of nuclear systems, especially those using thorium fuel, SMRs, and ways to combine nuclear power with renewable energy. New studies show advanced reactors can help create flexible, low‐carbon energy with better safety and lower costs. For example, current work focuses on linking nuclear systems with hydrogen production, heat storage, and grid integration. This makes them useful for more than just steady, basic electricity. Also, new studies comparing the costs and benefits show these advanced nuclear options can compete under changing climate rules and carbon pricing. These advances mark a significant shift from traditional nuclear power to more flexible, sustainable, and multi‐use energy systems.

## 2 Novelty and Aim of Study

Although there is a growing academic interest in sophisticated nuclear systems, a substantial deficit persists in the comprehensive evaluation of how alternative nuclear fuels and innovative reactor designs collectively tackle the essential challenges of sustainability, waste minimization, safety, and measures for non‐proliferation. The existing body of scholarly literature frequently investigates these dimensions disjointedly, overlooking the prospective synergistic interactions that could augment overall reactor efficacy and facilitate closed fuel cycle operations. Furthermore, the requisite policy, regulatory, and economic infrastructures essential for the commercial implementation of such systems have not been sufficiently examined.

This review intentionally integrates these facets by analyzing the roles of Thorium, uranium‐233, and minor actinides as alternative fuels in conjunction with MSRs, SMRs, and FBRs. Its objective is to thoroughly assess the technological advancements, ecological advantages, and obstacles to deployment associated with these innovations. By amalgamating insights from contemporary scholarly research, international pilot initiatives, and evolving policy frameworks, this study delineates a strategic framework for positioning nuclear energy as a sustainable cornerstone in future low‐carbon energy paradigms.

## 3 Alternative Nuclear Fuels

### 3.1 Thorium Fuel Cycle

Thorium‐232, recognized as a fertile isotope, has attracted considerable scholarly interest as a viable alternative to traditional uranium‐based nuclear fuels. A primary advantage of Thorium lies in its relative abundance, being approximately three to four times more prevalent than uranium within the Earth's crust [18]. Upon irradiation within a nuclear reactor, Thorium‐232 captures a neutron and transmits it into uranium‐233, a fissile substance capable of sustaining a nuclear chain reaction. This transmutation process underpins the operational framework of the Thorium fuel cycle [19]. The thorium fuel cycle offers several salient advantages. In contrast to the conventional uranium‐plutonium cycle, it produces significantly lower amounts of long‐lived radioactive waste, facilitating more straightforward waste management protocols [20]. Furthermore, Thorium engenders diminished quantities of plutonium and other transuranic elements, which exacerbate the radiotoxicity and long‐term risks associated with spent nuclear fuel [21]. Additionally, the uranium‐233 generated from Thorium is accompanied by minor quantities of uranium‐232, which emits highly penetrating gamma radiation. This property serves as a deterrent to the proliferation of nuclear weapons by rendering the material difficult to manage and shield [12].

Notwithstanding these advantages, the Thorium fuel cycle faces several notable obstacles. The advancement of reactors adept at efficiently harnessing Thorium, such as molten salt or heavy water reactors, necessitates substantial financial investment and specialized technical knowledge [22]. Moreover, the reprocessing of irradiated Thorium to recover uranium‐233 is complex due to the presence of highly radioactive isotopes. Nonetheless, ongoing research initiatives and pilot projects in nations such as India, China, and Norway are propelling the development of Thorium fuel technology [23].

### 3.2 Uranium‐233

Uranium‐233 is the fissile byproduct generated from the irradiation of Thorium‐232 and constitutes the fundamental basis of the Thorium fuel cycle. It demonstrates remarkable nuclear properties, including a substantial neutron yield per fission event and the capability to sustain chain reactions in both thermal and fast neutron environments [24]. This adaptability facilitates a variety of reactor design possibilities and enhances the neutron economy. Nevertheless, managing uranium‐233 entails notable technical and safety complications. Its concurrent generation with uranium‐232 raises significant radiation protection concerns attributable to intense gamma radiation emissions [25].

This situation necessitates the implementation of remote handling techniques and the utilization of specialized shielding during fuel fabrication and reprocessing. Furthermore, the extraction and purification of uranium‐233 from Thorium‐derived spent fuel requires sophisticated chemical processing technologies [26]. Despite these challenges, uranium‐233 continues to be regarded as a promising fuel source for innovative reactor designs. It presents opportunities for closed fuel cycles characterized by diminished environmental repercussions and improved resource efficiency. Ongoing research into uranium‐233‐powered reactor concepts persists, particularly within proliferation‐resistant and inherently safe systems [27].

### 3.3 Minor Actinides and Reprocessed Fuels

Minor actinides, including neptunium, americium, and curium, emerge as byproducts of traditional nuclear fuel cycles and play a crucial role in augmenting the long‐term radiotoxicity associated with spent nuclear fuel [28]. The incorporation of these isotopes into innovative fuel formulations has the potential to alleviate the burden on geological repositories while facilitating the transmutation of hazardous waste into isotopes with shorter half‐lives [2]. Reprocessing technologies, encompassing both aqueous and pyrochemical methodologies, enable the recovery and reutilization of fissile materials extracted from spent nuclear fuel. These methodologies facilitate the production of mixed oxide or actinide‐bearing fuel specifically designed for application in fast reactors or accelerator‐driven systems [29].

The implementation of minor actinide fuels encounters various technical and economic challenges. The management of highly radioactive substances, the development of compatible fuel matrices, and the assurance of reactor compatibility constitute active domains of ongoing research [30]. Nevertheless, the assimilation of reprocessed fuels into next‐generation reactors holds considerable promise for enhancing fuel efficiency and reducing the volume of nuclear waste generated [7]. Table 1 shows the comparative overview of alternative nuclear fuels.

**TABLE 1:** Comparative Overview of Alternative Nuclear Fuels [31, 32].

Fuel Type	Advantages	Challenges	Suitable Reactor Types	Deployment Status
Thorium‐232	Abundant, low‐waste, inherent proliferation resistance	Requires breeding, lacks industrial infrastructure	MSRs, Heavy Water Reactors, Fast Reactors	Pilot/demo in India, China, Norway
Uranium‐233	High neutron economy, flexible spectrum compatibility	Handling difficulties due to U‐232 contamination	MSRs, Advanced Thermal and Fast Reactors	Experimental; active R&D in India and China
Minor Actinides	Enables transmutation, reduces waste toxicity	High radiotoxicity, fabrication complexity	FBRs, ADS, MSRs	Lab‐scale research in EU, U.S., Japan
MOX Fuel (Pu + U)	Recycles plutonium, reduces fresh uranium requirement	Proliferation risk, reactor modification needed	LWRs, FBRs, Advanced Thermal Reactors	Commercial in France, limited use globally

## 4 Innovative Reactor Concepts

### 4.1 Molten Salt Reactors

MSRs represent a significant paradigm shift from traditional solid‐fuel reactors. They employ a liquid fuel formulation, typically comprising a solution of fissile substances dispersed within a molten salt medium [12]. Originally developed during the United States Aircraft Reactor Experiment and the MSRs experiment conducted at Oak Ridge National Laboratory, MSRs have witnessed a renewed interest due to their potential benefits in enhancing safety, optimizing fuel efficiency, and minimizing nuclear waste [33]. MSRs have been reported to achieve thermal efficiencies exceeding 45% at high temperatures (typically 600–700°C). However, these values depend on reactor configuration, salt composition, heat exchanger design, and system integration strategies. Variations in coolant chemistry and operational parameters can significantly influence achievable performance [34].

Operating at elevated temperatures and reduced pressures, MSRs substantially decrease the probability of explosive incidents while enhancing thermal efficiency to surpass 45%, in contrast to the 33%–35% efficiency typically observed in LWRs. The molten salt acts in dual capacity as both a vehicle for fuel transmission and as a coolant, thereby eliminating the requirement for solid fuel production and enabling the continuous removal of fission byproducts via online reprocessing. This characteristic allows MSRs to achieve higher fuel burnup rates while reducing the quantity of high‐level radioactive waste produced [35]. An additional significant advantage lies in their compatibility with Thorium‐based fuels. When incorporated into fluoride salts, Thorium can transmit to uranium‐233 within the reactor, thereby promoting a closed fuel cycle characterized by minimal plutonium generation [36]. The inherent negative temperature coefficient found in MSRs further ensures passive safety; as temperatures increase, the expansion of the fuel salt results in diminished reactivity, thereby mitigating the risk of overheating [37].

However, several technical challenges must be addressed. The corrosion resistance of structural materials subjected to high‐temperature fluoride or chloride salts constitutes a pivotal research focus [38]. Moreover, the chemical processing of molten salts and developing containment strategies for high‐temperature environments demand significant innovation [39]. Numerous startups and government‐backed initiatives, including the U.S. Department of Energy Advanced Reactor Demonstration Program, are actively overcoming these challenges and propelling MSRs toward commercial viability [9].

### 4.2 Small Modular Reactors

SMRs are a groundbreaking class of nuclear reactors meticulously designed for modular construction, reduced physical size, and adaptable deployment capabilities. Typically generating less than 300 MWe, SMRs offer a scalable alternative to conventional large‐scale reactors, making them especially beneficial for remote locations, industrial applications, and integrating renewable energy technologies [40]. The safety protocols embedded within SMRs utilize passive and inherent systems, reducing reliance on active cooling mechanisms and operator intervention. Various configurations of SMRs, such as NuScale Powers light‐water reactor module and the BWRX‐300, are engineered with features that guarantee walk‐away safety, permitting the dissipation of decay heat through natural convection processes. The factory‐based assembly of SMR components significantly shortens construction timelines, alleviates quality control issues, decreases capital costs, enhances cost predictability and the attractiveness of investment opportunities [41]. SMRs are widely recognized for their modular deployment and enhanced safety characteristics; however, their overall performance and economic viability depend on design standardization, manufacturing scale, and site‐specific deployment conditions. Variations in reactor type, such as light water, gas‐cooled, or fast reactors, result in differences in efficiency and operational flexibility [42].

In addition to the light‐water designs, advanced SMRs include high‐temperature gas‐cooled reactors, sodium‐cooled fast reactors and lead‐cooled fast reactors. Each is carefully engineered to deliver distinct operational advantages, such as higher outlet temperatures, closed fuel cycles, or improved load‐following capabilities [43]. The challenges confronting SMRs encompass the complexities of regulatory approval processes, which are primarily oriented towards large‐scale reactors, along with competitive pressures from economically viable renewable energy sources [44]. Establishing robust licensing frameworks and supply chains is crucial for the widespread adoption of SMRs. Nevertheless, the modular characteristics and favourable safety attributes of SMRs position them as a critical component in the evolution of future nuclear energy infrastructure [45].

### 4.3 Fast Breeder Reactors

FBRs utilize fast neutrons instead of the thermal neutron's characteristic of conventional reactors. This breeding mechanism transforms fertile isotopes, such as uranium‐238, into fissile materials like plutonium‐239 [46]. Consequently, this breeding mechanism allows FBRs to yield an excess of fuel compared to their consumption, thereby providing a pragmatic strategy for optimizing uranium resources while simultaneously alleviating the generation of nuclear waste [47]. FBRs typically employ liquid metal coolants, including sodium or lead, to achieve a superior neutron economy and efficient thermal management. Sodium‐cooled FBRs have been conceived and executed in several countries, including France (Phenix and Superphenix), Russia (BN series), and India (Prototype Fast Breeder Reactor) [48]. Such reactors can incinerate long‐lived actinides, significantly diminishing the radiotoxicity and thermal burden of nuclear waste designated for geological disposal [49].

Despite their intrinsic benefits, FBRs face many technological and economic obstacles. The chemical reactivity of liquid sodium with both atmospheric oxygen and water necessitates stringent safety measures [50]. Issues concerning operational reliability and cost‐efficiency remain, along with the complexities related to the breeding and reprocessing of plutonium. Nevertheless, advancements in fuel design, coolant chemistry, and reactor regulation are progressively improving the viability of FBRs [16]. FBRs are crucial in promoting a sustainable nuclear fuel cycle, enabling long‐term energy sustainability and waste reduction. They constitute an essential element of a holistic strategy in conjunction with MSRs and SMRs [3].

### 4.4 Hybrid and Multi‐Purpose Reactor Systems

Hybrid and multi‐functional nuclear reactors represent a modern framework that extends the utility of nuclear energy beyond the singular function of electricity generation. These systems are intricately designed to deliver various, versatile, and varied energy services [51]. These services include elevated‐temperature process heat for industrial applications, desalination, district heating, and hydrogen generation via high‐temperature electrolysis or thermochemical techniques [52]. For instance, TerraPower's Natrium reactor integrates a sodium‐cooled fast reactor with molten salt thermal energy storage to enable load‐following capabilities and cogeneration prospects [53]. Conversely, the European GEMINI+high‐temperature gas‐cooled reactor design is specifically optimized for the dual purpose of integrated power and hydrogen generation.

**FIGURE 1:**
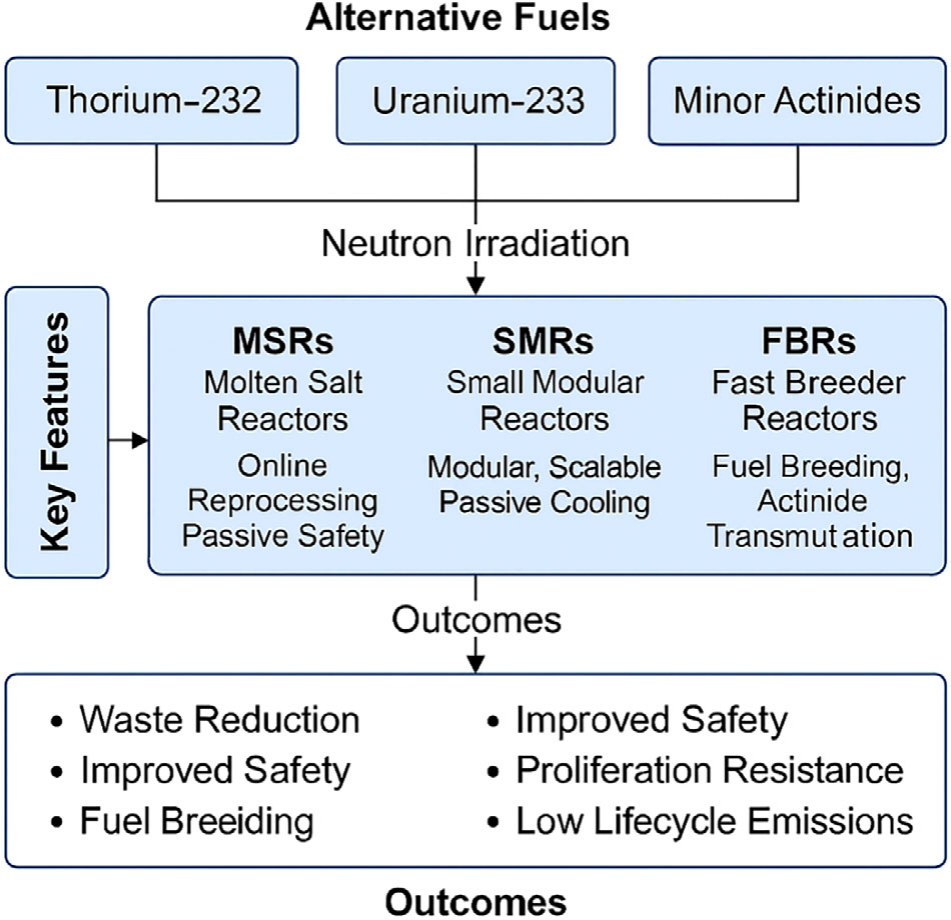
Integration of alternative fuels with advanced reactors for enhanced sustainability and safety in nuclear energy.

These multi‐functional reactors are engineered for optimal synergy with variable renewable energy sources by offering thermal buffering and rapid dispatch capabilities, thus contributing to improved grid stability within low‐carbon energy frameworks [54]. Although these systems are currently in the initial design or demonstration phases, they are receiving heightened interest due to their capacity to address sector‐coupling challenges associated with decarbonization initiatives [55]. However, their extensive deployment is dependent upon the resolution of critical challenges, including component durability under extreme operational conditions, compatibility of advanced materials, market readiness, and necessary regulatory modifications to accommodate hybrid energy outputs [56]. Figure 1 shows the integration of alternative fuels with advanced reactors for enhanced sustainability and safety in nuclear energy. Table 2 shows the comparative summary of reactor concepts.

**TABLE 2:** Comparative Summary of Reactor Concepts [10, 36].

Feature	MSR	SMR	FBR	Hybrid/multi‐purpose
Fuel Type	Liquid (Th‐U, U‐Pu salts)	Solid (LEU, MOX)	Solid (U‐Pu, Minor Actinides)	Various (salt, TRISO, MOX)
Coolant	Fluoride/Chloride salts	Light water/Gas/Sodium	Sodium/Lead	Depending on the system
Operating Temp (°C)	600–700	300–600	500–550	500–800
Safety Mechanism	Inherent, passive (freeze plug)	Passive (natural convection)	Feedback‐controlled	Multi‐layered (thermal + AI)
Fuel Utilization	Very high (online reprocessing)	Medium (batch cycle)	High (breeding >1)	Flexible (depending on configuration)
Waste Output	Low	Moderate	Low (transmutation capable)	Variable
Proliferation Resistance	High (U‐232 barrier)	Moderate	Moderate	High with closed fuel cycles
TRL	5–6	7–8	7–8	4–6
Deployment Readiness	Pilot	Near commercial	Demonstration/Operational	Early development

A comparative evaluation of MSRs, SMRs, and FBRs shows clear performance and deployment trade‐offs. MSRs offer high thermal efficiency and better fuel use but face material and chemical processing challenges. SMRs are suited for early deployment thanks to modular builds and passive safety; their economics depend on mass production. FBRs maximize resource efficiency and cut waste through breeding and actinide transmutation, but their complex operation and liquid‐metal coolant safety issues are substantial drawbacks. These reactors should be seen as complementary options in a broad low‐carbon nuclear portfolio, not as direct rivals.

## 5 Technological and Economic Considerations

Deploying sustainable nuclear energy systems, particularly those incorporating alternative fuels and advanced reactor designs, require considerable advancements in core technology, supporting infrastructure, economic structures, and lifecycle integration methodologies [57]. This segment elucidates emerging nuclear systems' technological readiness, production requirements, economic barriers, and lifecycle advantages [18].

### 5.1 Fuel Fabrication and Supply Chain Modernization

The incorporation of Thorium‐based fuels, specifically uranium‐233 and materials derived from actinide recycling, engenders significant modifications to the fuel cycle infrastructure. Traditional uranium oxide fabrication facilities are inadequately equipped to handle materials characterized by elevated radiotoxicity or non‐standard fuel architectures [31]. The conversion of Thorium into Thorium dioxide is typically a prerequisite, with the subsequent formation of pellets or utilization in a dissolved state within MSRs [58]. Uranium‐233, produced through the neutron irradiation of Thorium‐232, poses unique challenges in handling due to its simultaneous generation alongside uranium‐232, a formidable gamma emitter that requires remote processing and shielding protocols [59]. Similarly, fuel compositions incorporating trace amounts of actinides like neptunium, americium, and curium demand advanced containment systems and automation due to their heightened thermal output and radiotoxicity [60].

Developing corrosion‐resistant alloys, high‐temperature containment systems, and specialized reprocessing facilities is crucial for effectively managing molten salt fuels and actinide‐rich materials. To establish resilient supply chains, it is essential to advance strategic procurement of Thorium and expand processing methodologies, including pyrochemical and aqueous partitioning techniques [21]. Furthermore, international collaboration regarding fuel qualification and the regulatory framework governing material transportation will be indispensable for the global adoption of these innovative technologies [61].

### 5.2 Capital and Operating Cost Analysis

Although advanced nuclear reactors can significantly enhance fuel efficiency and safety, their inherently capital‐intensive nature remains limited. The early phases of MSRs, SMRs, and FBRs involve significant financial implications associated with development and regulatory adherence [62]. While the process of modularisation can reduce both the duration of on‐site construction and the requisite labour force, it is imperative that these systems are meticulously designed to incorporate multiple units at each location to realize economies of scale [63]. The competitiveness of lifecycle cost assessments is significantly bolstered when one considers the ramifications of reduced refuelling intervals, increased burnup rates, and the minimization of waste management obligations [64]. Financial innovations, encompassing a range of instruments such as green bonds, government‐backed insurance mechanisms, and their integration within national climate taxonomies, can effectively promote the successful implementation of such systems [65]. Table 3 shows the estimated CAPEX and economic attributes of advanced nuclear systems.

**TABLE 3:** Estimated CAPEX and economic attributes of advanced nuclear systems [42, 66, 67, 68].

Reactor Type	Estimated CAPEX (USD/kW)	LCOE (USD/MWh)	Key Economic Feature
SMR (e.g., NuScale, BWRX‐300)	4000–6000	70–90	Modular deployment, reduced on‐site labor
MSR	7000–9500	75–95	High thermal efficiency, continuous reprocessing
FBR	6000–8000	65–85	Fuel breeding, long core life

### 5.3 Waste Management and Lifecycle Integration

Advanced nuclear fuel cycles demonstrate significant enhancements in the characteristics of radioactive waste. Fast neutron reactor systems can transmute isotopes characterized by extended half‐lives, while MSRs enable continuous reprocessing and advancements in fuel burnup efficiency [69]. These sophisticated functionalities reduce the volume of spent nuclear fuel, its concomitant radiotoxicity, and the spatial demands for waste repositories [70].

The overall lifecycle greenhouse gas emissions associated with nuclear energy, which incorporate stages such as construction, mining, operational activities, and decommissioning, are consistently kept below 12 gCO_2_e/kWh, a value that is analogous to that of wind energy and markedly lower than that emitted by solar photovoltaics and fossil fuel sources [71]. Moreover, fuel cycles predicated on Thorium and actinides alleviate environmental repercussions by diminishing the necessity for mining activities and enhancing fuel efficiency. These systems additionally facilitate the transition towards comprehensive lifecycle management, in which nuclear operations are incorporated within circular resource frameworks and low‐emission supply chains [72]. Table 4 shows the waste reduction and environmental advantages of advanced reactor systems.

**TABLE 4:** Waste reduction and environmental advantages of advanced reactor systems [15, 73, 74].

System	Waste VOLUME	Waste Heat Load	Radiotoxicity	Key Waste Strategy
MSR	Low (in situ reprocessing)	Low	Reduced due to continuous fission	Online salt cleanup
SMR	Moderate	Moderate	Standard SNF profile	Dry storage or reprocessing
FBR	Low (actinide transmutation)	Low	Transmutation of minor actinides	Closed fuel cycle

### 5.4 Deployment Timelines and Commercial Readiness

The commercial readiness of advanced nuclear technologies differs significantly across reactor categories. For instance, SMRs are nearing market entry in multiple regions, facilitated by ongoing licensing and demonstration initiatives [75]. In contrast, MSRs and FBRs predominantly remain at the pilot or demonstration stage, with most deployments expected after 2030. Variations in regulatory frameworks, investment risks, and technological maturity further contribute to these divergent development timelines [76]. Table 5 provides deployment status of selected advanced reactor technologies.

**TABLE 5:** Deployment status of selected advanced reactor technologies [77, 78].

Reactor Type	FOAK (first‐of‐a‐kind) Timeline	Current Status	Commercial Readiness
SMR	2025–2028	Regulatory licensing (e.g., NRC, CNSC)	Medium (pilot‐ready)
MSR	2030+	R&D and demonstration stage (e.g., TMSR‐LF1, Terrestrial Energy)	Low to medium
FBR	Ongoing (BN‐800, PFBR)	Operational in Russia and India	Medium (state‐backed)

## 6 Environmental and Safety Impacts

### 6.1 Lifecycle Emissions

**FIGURE 2:**
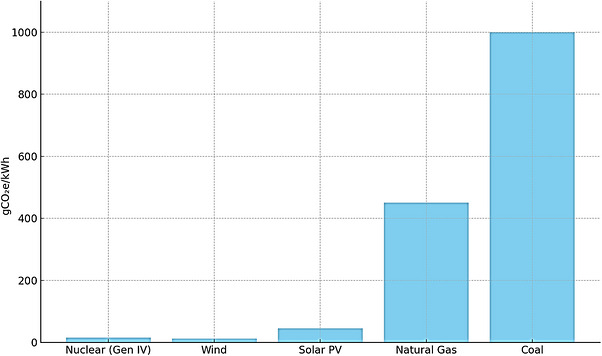
Lifecycle GHG emissions of various energy sources (gCO_2_e/kWh).

Nuclear energy exhibits some of the lowest lifecycle GHG emissions compared to all power generation technologies, with estimates ranging between 12–20 gCO_2_e/kWh. These estimates encompass a comprehensive array of activities, which include mining, fuel processing, construction, operational phases, and decommissioning processes [79]. Advanced reactor technologies such as MSRS and FBRS enhance sustainability by enabling superior fuel burnup, which necessitates lower enrichment levels, and facilitate closed fuel cycles that reduce the demand for resource extraction while minimizing the production of radioactive waste [80]. The application of Thorium‐based fuels further refines the emissions profile due to their relative natural abundance and the opportunity for localized sourcing. Implementing on‐site reprocessing alongside a reduction in material throughput substantially aids in mitigating the environmental impact of nuclear energy across its entire lifecycle [81]. Figure 2 shows the lifecycle GHG emissions of various energy sources.

### 6.2 Safety Enhancements

**FIGURE 3:**
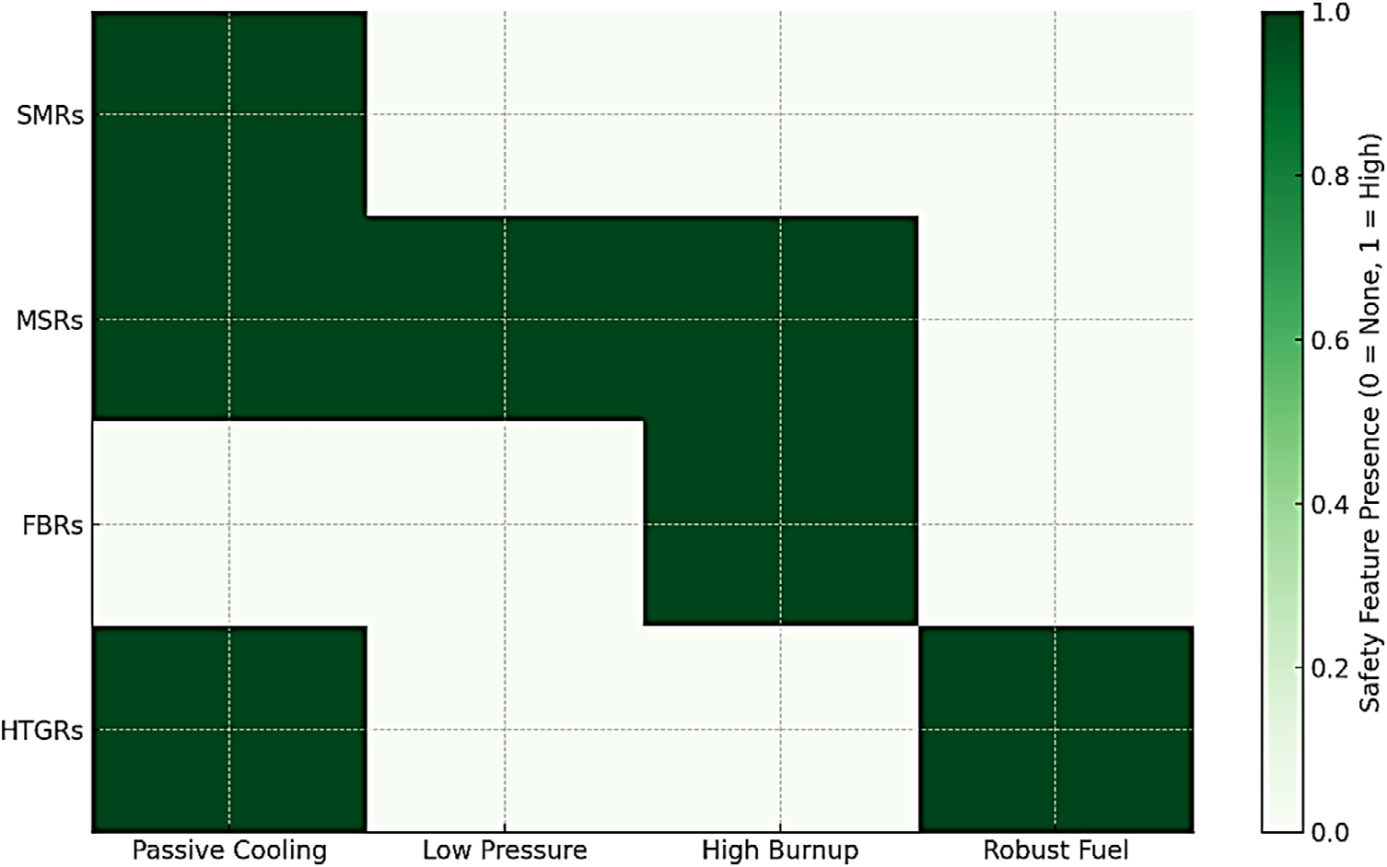
Comparison of safety features across advanced reactor types.

Next‐generation nuclear reactors are engineered with intrinsic and passive safety features that mitigate the risk of accidents without necessitating operator intervention or reliance on external power sources [82]. MSRs are characterized by their low‐pressure operational protocols and freeze plug mechanisms that facilitate fuel drainage in overheating instances. In contrast, SMRs employ compact architecture and gravity‐driven cooling systems to manage decay heat adeptly [83]. FBRs utilize liquid metal coolants such as sodium or lead, which promote efficient thermal transfer and enhance reactor stability. Innovative fuel formulations, exemplified by TRISO (TRi‐structural ISOtropic) particles, provide exceptional containment of fission byproducts even under extreme thermal conditions [84]. Furthermore, incorporating real‐time monitoring and sophisticated digital control systems markedly diminishes the likelihood of human error, thereby ensuring elevated operational safety standards [43]. Figure 3 shows the comparison of safety features across advanced reactor types.

### 6.3 Proliferation Resistance

**FIGURE 4:**
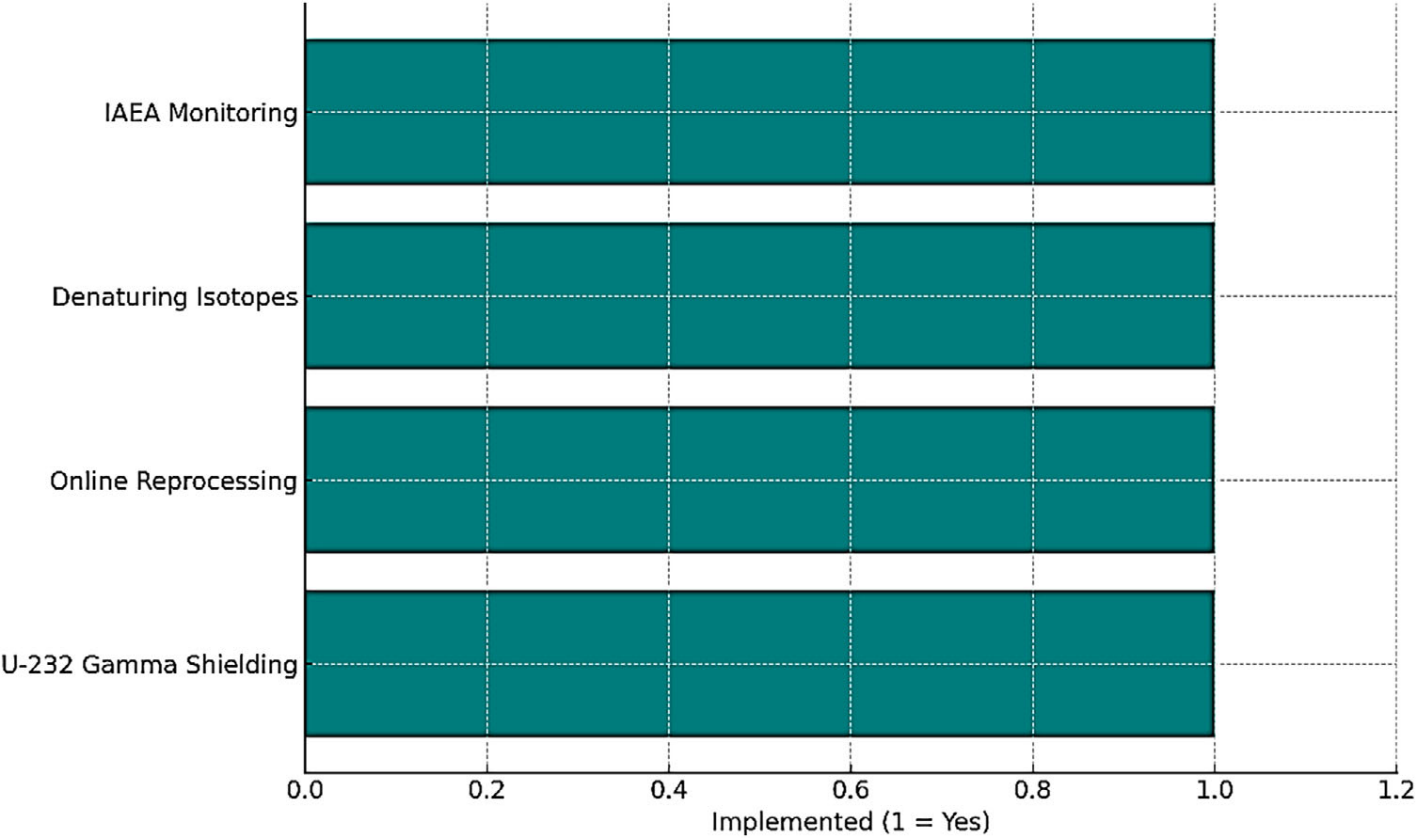
Proliferation resistance strategies in modern nuclear systems.

The resistance to proliferation is enhanced in alternative fuel cycles and advanced reactor configurations through the intrinsic properties of materials and the overarching architecture of the systems [25]. Thorium‐uranium fuel cycles intrinsically deter the development of nuclear weapons owing to the concurrent production of uranium‐232, a radionuclide that emits potent gamma radiation, thereby rendering the handling and diversion of materials exceedingly challenging [26]. MSRs inhibit the accumulation of isotopes that can be utilized for weapons by facilitating continuous fuel reprocessing and incorporating denaturants within their operational framework. Fast reactors operating within closed fuel cycles can integrate co‐processing, thus complicating the separation of isotopes [85]. The amalgamation of these technical characteristics, robust international safeguards, real‐time monitoring methodologies, and stringent material accounting protocols significantly enhances the efficacy of nuclear non‐proliferation initiatives [86]. Figure 4 shows proliferation resistance strategies in modern nuclear systems.

## 7 Policy and Regulatory Framework

The practical implementation of sustainable nuclear technologies is contingent not solely upon technical preparedness and economic feasibility but also on establishing extensive policy and regulatory frameworks. Such frameworks must promote innovation, guarantee safety, facilitate financing mechanisms, and be congruent with international climate goals and energy transitions.

### 7.1 Modernizing Regulatory Processes

The current regulatory frameworks are predominantly designed for traditional LWRs and frequently fall short in their capacity to evaluate advanced reactor technologies that employ innovative fuels, passive safety mechanisms, and modular architecture [87]. Such inadequacies result in considerable delays for emerging technologies, including MSRs and SMRs, which deviate from established design paradigms [88]. Regulatory bodies, including the U.S. Nuclear Regulatory Commission and the Canadian Nuclear Safety Commission, are increasingly implementing risk‐informed, performance‐based licensing methodologies to promote technological innovation [89]. These strategies prioritize safety outcomes and probabilistic risk assessments over rigid compliance standards. Tools for early engagement, such as pre‐licensing consultations, vendor design evaluations, and regulatory sandboxes, are being employed more frequently to expedite the approval processes and mitigate uncertainties throughout the technology development lifecycle [78].

On an international scale, initiatives such as the International Atomic Energy Agency's (IAEA) Nuclear Harmonisation and Standardisation Initiative and the Generation IV International Forum are advocating for regulatory alignment to facilitate technology transfer and collaborative deployment. Establishing harmonized safety standards across national boundaries will be crucial for fostering international cooperation and expediting the commercial rollout of advanced reactor technologies [90].

### 7.2 Supporting Research, Development, and Demonstration

Investment in research, development, and demonstration is imperative to expedite advancements in reactor design, fuel cycle technologies, material durability, and hybrid applications [91]. Government‐sponsored initiatives such as the U.S. Department of Energy's Advanced Reactor Demonstration Program and the European Sustainable Nuclear Industrial Initiative furnish essential funding and infrastructural support for nascent deployment stages [92]. Collaborative RD&D initiatives among national laboratories, academic institutions, private enterprises, and international coalitions are vital for mitigating innovation risks and disseminating knowledge [93]. Demonstration projects, including TerraPower's Natrium reactor and X‐Energy Xe‐100 SMRs in the United States, as well as China's Thorium‐based TMSR‐LF1, are instrumental in corroborating the technical and economic viability of next‐generation systems. First‐of‐a‐kind deployments frequently necessitate supplementary risk mitigation strategies [94]. Such strategies encompass regulatory sandboxes, technology‐inclusive licensing frameworks, and innovative financing mechanisms that involve public‐private partnerships to facilitate prompt commercialization [95].

### 7.3 Public Engagement and Risk Communication

Public perception exerts a considerable influence on the efficacy of nuclear initiatives. Misunderstandings regarding radiation, nuclear incidents, and waste management frequently exacerbate opposition and hinder implementation [96]. Consequently, the adoption of transparent and inclusive risk communication methodologies is imperative. Effective public engagement encompasses providing open‐access information about reactor safety and ecological performance, organizing public consultations, and enhancing scientific literacy through educational programs [97]. The community's involvement in site selection processes and the establishment of benefit‐sharing frameworks can further cultivate social licenses. Emphasizing the environmental benefits of advanced nuclear technology, such as reduced emissions, minimal land requirements, and improved safety features, can transform public discourse and promote acceptance [98].

### 7.4 Integration with Climate and Energy Policy

Nuclear energy integration within comprehensive climate and energy frameworks is imperative to capitalize on its potential for decarbonization fully. Nationally Determined Contributions, as delineated in the Paris Agreement, ought to explicitly acknowledge nuclear energy as a zero‐carbon energy source capable of supplanting fossil fuels in baseload and industrial applications [1]. Incorporating nuclear technologies into national and regional clean energy taxonomies, such as the European Union's Green Taxonomy and India's climate finance policy, facilitates access to sustainable financing and market‐driven incentives [57].

Nuclear energy engagement in carbon pricing mechanisms, clean energy credit frameworks, and green bond markets can further enhance its competitive standing [99]. Moreover, nuclear energy integration with renewable energy systems and non‐electric applications is essential. This encompasses grid balancing, cogeneration, heat supply, and hydrogen generation. Cross‐sectoral collaboration among energy, environmental, industrial, and transportation agencies is vital to optimizing nuclear energy's role within a sustainable and resilient energy infrastructure [100].

## 8 Challenges and Future Directions

Advanced nuclear pathways offer significant potential but face technical and economic constraints. Key technical challenges include materials degradation under extreme conditions, the complex handling and fabrication of fuels like uranium‐233 and minor actinides, and the need for advanced reprocessing infrastructure. Current regulatory frameworks do not align with innovative reactor designs, slowing licensing. High upfront costs, long construction times, and uncertain returns hinder commercialization. Immature supply chains and limited economies of scale reduce cost competitiveness versus other low‐carbon technologies. Widespread adoption of next‐generation nuclear systems depends on technological innovation, regulatory reform, and financial risk‐sharing.

### 8.1 Corrosion and Materials Challenges in MSRs

MSRs offer distinctive advantages regarding thermal efficiency and the capability for online reprocessing; however, their path to commercialization is significantly obstructed by formidable materials‐related challenges. The high‐temperature fluoride and chloride salts employed in MSRs exhibit pronounced corrosive properties, particularly when irradiation, resulting in accelerated deterioration of conventional structural alloys [101]. The advancement of sophisticated corrosion‐resistant materials, including nickel‐based alloys, silicon carbide composites, and cutting‐edge protective coatings, is paramount. Moreover, adopting real‐time corrosion monitoring technologies will be imperative to guarantee the long‐term integrity and safety of the reactor [10].

### 8.2 Fuel Handling and Fabrication Complexities

The management and production of alternative fuels, including uranium‐233 and minor actinides, engender multifaceted operational challenges. Uranium‐233, which is synthesized from Thorium, is consistently tainted with uranium‐232, a radionuclide that emits penetrating gamma radiation, thereby necessitating the implementation of shielded remote handling systems [58]. Minor actinides such as neptunium and americium exhibit significant radiotoxicity and produce considerable thermal output, complicating their integration into stable fuel matrices. Progress in automated fuel fabrication, remote processing technologies, and additive manufacturing will be essential for safely and efficiently utilizing these fuel types in the next generation of reactors [35].

### 8.3 Regulatory Bottlenecks and Licensing Reform

Regulatory frameworks currently exhibit a significant lag advancement in technological innovation, particularly concerning reactor designs that deviate from conventional LWRs. The existing pathways for licensing are characterized by their protracted timelines, inflexible nature, and inadequacy in evaluating reactors equipped with passive safety mechanisms, modular components, or innovative fuel cycles [89]. To address these impediments, national and international regulatory authorities must implement licensing strategies that include technology and are informed by risk, emphasizing performance outcomes strongly [102]. Efforts toward international harmonization, exemplified by initiatives led by the IAEA, alongside the advancement of model‐based digital licensing instruments, will play a crucial role in expediting the approval processes for reactors while ensuring the maintenance of stringent safety standards [103].

### 8.4 Economic Viability and FOAK Deployment Risks

From an economic perspective, introducing FOAK reactors presents considerable risks attributable to elevated capital expenditures, protracted construction timelines, and ambiguous returns on investment. These obstacles are particularly accentuated for MSRs and FBRs, necessitating specialized infrastructure and fuel cycles [41]. The establishment of public‐private partnerships, the implementation of cost‐sharing frameworks, and the formulation of supportive governmental policies are imperative to mitigate risks associated with initial projects [65]. Furthermore, adopting factory modularisation and utilizing standardized supply chains can contribute to reducing construction duration and costs. At the same time, exploring multi‐purpose applications such as hydrogen production and industrial process heat can enhance the economic justification for deployment [33].

### 8.5 Public Perception and Social License

Public acceptance continues to present a significant challenge for advancing nuclear energy. Historical incidents and enduring apprehensions regarding radiation, waste management, and safety protocols have significantly influenced public dissent in numerous regions. Implementing proactive public engagement strategies is imperative to mitigate this opposition [104]. Transparent communication regarding risks, availability of open‐access data, systematic public consultations, and comprehensive educational initiatives can enhance scientific literacy and foster community trust. Engaging local populations in site selection processes and providing shared economic advantages will further augment social license and acceptance of projects [105].

### 8.6 Integration with Renewable‐Based Energy Systems

Ultimately, incorporating sophisticated nuclear reactors into energy systems predominantly reliant on variable renewable sources necessitates substantial innovation. Prospective reactors must demonstrate adaptable, load‐following capabilities to effectively manage the variability inherent in wind and solar energy production [106]. Hybrid configurations integrating nuclear energy storage, thermal buffering, or hydrogen synthesis are viable for establishing resilient, low‐carbon energy infrastructures. Investigation into advanced control algorithms, innovative grid interfaces, and sector coupling is paramount for actualizing this vision and optimizing the contribution of nuclear energy within a comprehensively decarbonized future [107]. Table 6 shows the future challenges and strategic directions in nuclear sustainability.

**TABLE 6:** Future Challenges and Strategic Directions in Nuclear Sustainability [108, 109, 110].

Challenge Area	Issues	Future Directions
Corrosion and Materials in MSRs	Salt‐induced corrosion at high temperatures	Develop corrosion‐resistant alloys and coatings; implement in‐situ corrosion monitoring.
Fuel Handling and Fabrication	U‐232 gamma emission; radiotoxicity of minor actinides	Remote fuel processing, advanced shielding, automation, and additive manufacturing
Regulatory Bottlenecks	LWR‐based regulatory systems unsuitable for new designs	Risk‐informed, performance‐based licensing; digital twin‐enabled safety assessments
Economic Viability of FOAK Reactors	High CAPEX, construction delays, investor risk	Public‐private models, modularization, multi‐product systems (e.g., heat, hydrogen)
Public Acceptance and Social License	Misconceptions about safety, radiation, and waste	Transparent communication, stakeholder engagement, education, and benefit‐sharing
Integration with Renewable Energy Systems	Nuclear inflexibility for variable renewables	Flexible reactor controls, thermal/hydrogen storage, smart grid integration

## 9 Conclusions

Advanced nuclear energy systems utilizing alternative fuels such as thorium and uranium‐233, and innovative reactor designs such as MSRs, SMRs, and FBRs present significant opportunities for low‐carbon, resource‐efficient energy generation. The main argument is that these technologies can improve fuel utilization, enhance safety, and reduce the generation of long‐lived radioactive waste compared with conventional nuclear systems.

However, advancing these promising nuclear technologies to large‐scale deployment hinges on overcoming several technical, economic, and regulatory challenges that impede their commercial adoption. Specifically, unresolved issues include material durability, fuel‐handling complexities, licensing frameworks, and high initial capital costs, as well as uncertainties regarding public acceptance and policy support.

Currently, while certain technologies, especially SMRs, are close to commercial readiness, others, such as MSRs and advanced closed fuel cycles, remain in earlier development phases. The main argument holds only if ongoing research, pilot‐scale validation, and supportive policy mechanisms successfully lead to integration of these technologies into global energy portfolios.

In summary, to achieve decarbonization objectives, advanced nuclear technologies could play a pivotal complementary role alongside renewable energy. Fully realizing this potential requires a coordinated approach that prioritizes technological innovation, regulatory adaptation, and active stakeholder engagement together form the core of the main argument for their adoption.

## Conflicts of Interest

The authors declare no conflicts of interest.

## Declaration of Generative AI

To enhance the intelligibility and language of this work, the authors implemented ChatGPT as an AI‐assisted technology. After utilizing this tool/service, the authors reviewed and edited the content as necessary and accepted full responsibility for the publication's content.

## Data Availability

The data supporting this study's findings are available from the corresponding author upon reasonable request.

## References

[1] J. Parsons , J. Buongiorno , M. Corradini , and D. Petti , “A Fresh Look at Nuclear Energy,” Science 363, no. 6423 (2019): 105, 10.1126/science.aaw5304. 30630905

[2] A. Verbruggen and Y. Yurchenko , “Poitioning Nuclear Power in the Low‐Carbon Electricity Transition,” Sustainability 9, no. 1 (2017): 163, 10.3390/su9010163.

[3] R. Jayabal , “Next‐Generation Solutions for Water Sustainability in Nuclear Power Plants: Innovations and Challenges,” Nuclear Engineering and Design 432 (2025): 113757, 10.1016/j.nucengdes.2024.113757.

[4] A. Cho , “Risky Reactor Fuel to Linger,” Science 351, no. 6273 (2016): 544, 10.1126/science.351.6273.544. 26912677

[5] M. V. Ramana , “Technical and Social Problems of Nuclear Waste,” Wiley Interdisciplinary Reviews: Energy and Environment 7, no. 4 (2018): 289, 10.1002/wene.289.

[6] M. I. Ojovan , “Nuclear Waste Disposal,” Environmental Science & Technology 16, no. 12 (2023): 653A, 10.1021/es00106a723. 22646889

[7] S. S. Mustafa , “Improving the Reactor Safety Aspects by the Implementation of (Th‐U233‐Pu) Fuel in a PWR Assembly,” Nuclear Engineering and Design 422 (2024): 113147, 10.1016/j.nucengdes.2024.113147.

[8] M. Huo , Z. Fan , J. Qi , N. Qi , and D. Zhu , “Fast Analysis of Multi‐Asteroid Exploration Mission Using Multiple Electric Sails,” Journal of Guidance, Control, and Dynamics 46, no. 5 (2023): 1015–1022, 10.2514/1.G006972.

[9] B. Zhang , Y. Xue , Y. Zhang , K. Su , et al., “A Self‐Calibrating Digital Twin for Space Thermionic Nuclear Reactors under Fault Conditions,” Reliability Engineering & System Safety 268 (2025): 111995, 10.1016/j.ress.2025.111995.

[10] K. Carlson , L. Gardner , J. Moon , B. Riley , J. Amoroso , and D. Chidambaram , “Molten Salt Reactors and Electrochemical Reprocessing: Synthesis and Chemical Durability of Potential Waste Forms for Metal and Salt Waste Streams,” International Materials Reviews 66, no. 5 (2021): 339–363, 10.1080/09506608.2020.1801229.

[11] M. Wen , J. Han , W. Li , X. Chang , Q. Chu , and D. Chen , “EMFF‐2025: A General Neural Network Potential for Energetic Materials with C, H, N, and O Elements,” npj Computational Materials 11, no. 1 (2025): 333, 10.1038/s41524-025-01809-w.

[12] V. Pershukov , V. Artisyuk , A. Kashirsky , et al., “Paving the Way to Green Status for Nuclear Power,” Sustainability 14, no. 15 (2022): 9339, 10.3390/su14159339.

[13] G. H. Soto and X. Martinez‐Cobas , “Nuclear Energy Generation's Impact on the CO_2_ Emissions and Ecological Footprint Among European Union Countries,” Science of The Total Environment 945 (2024): 173844, 10.1016/j.scitotenv.2024.173844. 38871309

[14] Z. Drace , M. I. Ojovan , S. K. Samanta , et al., “Challenges in Planning of Integrated Nuclear Waste Management,” Sustainability 14, no. 21 (2022): 14204, 10.3390/su142114204.

[15] L. Rodriguez‐Penalonga , B. M. Soria , et al., “A Review of Nuclear Fuel Cycle Strategies and Spent Nuclear Fuel Management Technologies,” Energies 10, no. 8 (2017): 1235, 10.3390/en10081235.

[16] D. He , H. Li , R. Gao , et al., “INSL‐FLASH: A Fission Response Function Based Low‐Cost Adaptive Reactor Physics Code for High‐Fidelity Simulation,” Nuclear Science and Engineering 199 1 (2025): 1–19, 10.1080/00295639.2025.2561327.

[17] J. Serp , C. Poinssot , S. Bourg , et al., “Assessment of Environmental Footprint of Future Nuclear Energy Systems,” Energies 10, no. 9 (2017): 1445, 10.3390/en10091445.

[18] F. Hu , H. Yang , S. Wei , H. Zhou , Y. Chen , and H. Hu , “Urban Green Technology Transfer Networks and Green Finance Development: Evidence from the Yangtze River Delta, China,” Pacific‐Basin Finance Journal 96 (2026): 103055, 10.1016/j.pacfin.2026.103055.

[19] C. Selvam D , Y. Devarajan , and T. Raja , “Potential Benefits of Utilizing Nuclear Waste for Biodiesel Production,” Kerntechnik 89, no. 3 (2024): 368–381, 10.1515/kern-2024-0010.

[20] T. Chenniappan and Y. Devarajan , “Surface Contamination in Nuclear Wastewater: Identification and Mitigation,” Kerntechnik 89 (2024): 70, 10.1515/kern-2024-0070.

[21] R. Thandavamoorthy and Y. Devarajan , “Nuclear Shielding Behavior of Basalt/Carbon fiber Composites,” Nuclear Engineering and Technology 57 (2024): 103200, 10.1016/j.net.2024.09.003.

[22] F. Hu , H. Yang , X. Zhou , et al., “Pollution Transfer and Environmental Health Implications: Network Evolution and Proximity Mechanisms in the Yangtze River Delta, China,” Frontiers in Public Health 14 (2026): 1770901, 10.3389/fpubh.2026.1770901. 41778135 PMC12950677

[23] A. Schwenk‐Ferrero and A. A. Andrianov , “Comparison of Nuclear Fuel Cycle Options for Sustainable Performance,” Sustainability 9, no. 9 (2017): 1623, 10.3390/su9091623.

[24] B.‐S. Yu , G.‐C. Chen , H. Zhang , W.‐T. Cao , G.‐Y. Tang , and X. Tao , “Updated and Revised Neutron Reaction Data for 233 U,” Chinese Physics C 37, no. 7 (2013): 074001, 10.1088/1674-1137/37/7/074001.

[25] O. Searfus , P. Marleau , E. Uribe , H. Reedy , and I. Jovanovic , “Passive and Active Neutron Signatures of 233U for Nondestructive Assay,” Physical Review Applied 20 (2023): 064038, 10.1103/physrevapplied.20.064038.

[26] V. Jagannathan , “Thorium Breeder Reactors as a Power Source for 21st Century and Beyond,” International Journal of Energy Research 42, no. 1 (2018): 117–133, 10.1002/er.3951.

[27] L. He , L. Chen , S. Xia , and Y. Zou , “Minor Actinides Transmutation and 233U Breeding in a Closed Th‐U Cycle Based on Molten Chloride Salt Fast Reactor,” Energies 15, no. 24 (2022): 9472, 10.3390/en15249472. PMC9738889 36500054

[28] S. F. Ashley , G. T. Parks , W. J. Nuttall , C. Boxall , and R. W. Grimes , “Thorium Fuel Has Risks,” Nature 492, no. 7427 (2012): 31–33, 10.1038/492031a. 23222590

[29] S. A. Ansari , P. Pathak , P. K. Mohapatra , and V. K. Manchanda , “Aqueous Partitioning of Minor Actinides by Different Processes,” Separation & Purification Reviews 40, no. 1 (2011): 43–76, 10.1080/15422119.2010.545466.

[30] S. van Til , P. R. Hania , A. V. Fedorov , et al., “Irradiation Performance and First Examinations of Americium Bearing Blanket Fuel from the MARINE Irradiation Experiment,” Journal of Nuclear Materials 587 (2023): 154699, 10.1016/j.jnucmat.2023.154699.

[31] U. E. Humphrey and M. U. Khandaker , “Viability of Thorium‐based Nuclear Fuel Cycle for the next Generation Nuclear Reactor: Issues and Prospects,” Renewable and Sustainable Energy Reviews 97 (2018): 259–275, 10.1016/j.rser.2018.08.019.

[32] R. Akbari , M. A. Nasr , F. D'Auria , A. Cammi , J. R. Maiorino , and G. L. de Stefani , “Thorium‐Transuranic Fuel Deployment in SMRs,” Nuclear Engineering and Design 421 (2024): 113090, 10.1016/j.nucengdes.2024.113090.

[33] R. Luo , C. Liu , and R. Macián‐Juan , “Investigation of Control Characteristics for a Molten Salt Reactor Plant Under Normal and Accident Conditions,” Energies 14, no. 17 (2021): 5279, 10.3390/en14175279.

[34] D. Zhang , L. Liu , M. Liu , et al., “Review of Conceptual Design and Fundamental Research of Molten Salt Reactors in China,” International Journal of Energy Research 42, no. 5 (2018): 1834–1848, 10.1002/er.3979.

[35] C. A. M. Silva , I. R. Magalhães , M. Lorduy‐Alós , et al., “A Neutronic Evaluation of a Thorium‐based Molten Salt Breeder Reactor,” Nuclear Engineering and Design 421 (2024): 113049, 10.1016/j.nucengdes.2024.113049.

[36] O. Noori‐kalkhoran , L. Jain , and B. Merk , “On the Use of a Chloride or Fluoride Salt Fuel System in Advanced Molten Salt Reactors, Part 3; Radiation Damage,” Energies 17 (2024): 4772, 10.3390/en17061475.

[37] C. Yu , J. Wu , C. Zou , X. Cai , Y. Ma , and J. Chen , “Thorium Utilization in a Small Modular Molten Salt Reactor with Progressive Fuel Cycle Modes,” International Journal of Energy Research 43, no. 8 (2019): 3628–3639, 10.1002/er.4511.

[38] N. S. Patel , V. Pavlík , and M. Boca , “High‐Temperature Corrosion Behavior of Superalloys in Molten Salts – A Review,” Critical Reviews in Solid State and Materials Sciences 42, no. 1 (2017): 83–97, 10.1080/10408436.2016.1243090.

[39] D. Jiang , D. Zhang , X. Li , et al., “Fluoride‐salt‐cooled High‐temperature Reactors: Review of Historical Milestones, Research Status, Challenges, and Outlook,” Renewable and Sustainable Energy Reviews 161 (2022): 112345, 10.1016/j.rser.2022.112345.

[40] J. K. Nøland , M. Hjelmeland , L. B. Tjernberg , and C. Hartmann , “The Race to Realize Small Modular Reactors: Rapid Deployment of Clean Dispatchable Energy Sources,” IEEE Power and Energy Magazine 22 (2024): 90–103, 10.1109/mpe.2024.3357468.

[41] E. Shobeiri , F. Genco , D. Hoornweg , and A. Tokuhiro , “Small Modular Reactor Deployment and Obstacles to be Overcome,” Energies 16, no. 8 (2023): 3468, 10.3390/en16083468.

[42] J. K. Nøland , M. Hjelmeland , C. Hartmann , L. B. Tjernberg , and M. Korpas , “Overview of Small Modular and Advanced Nuclear Reactors in Energy Transition,” IEEE Transactions on Energy Conversion 40 (2025): 1–12, 10.1109/tec.2025.3529616.

[43] M. Cooper , “Small Modular Reactors and the Future of Nuclear Power in the United States,” Energy Research & Social Science 3 (2014): 161–177, 10.1016/j.erss.2014.07.014.

[44] S. Wei , H. Sun , C. Wang , et al., “Neutronic/Thermal‐Hydraulic Design Features of an Improved Lead‐Bismuth Cooled Small Modular Fast Reactor,” International Journal of Energy Research 43, no. 8 (2019): 3794–3805, 10.1002/er.4541.

[45] V.‐K. Hoang , G. T. T. Phan , V.‐P. Tran , et al., “Core Design Optimization of a 200 MWt Pressurized Water SMR Using Evolutionary Simulated Annealing,” Nuclear Engineering and Design 418 (2024): 112892, 10.1016/j.nucengdes.2023.112892.

[46] T. Wakabayashi , “Concept of a Fast Breeder Reactor to Transmute MAs and LLFPs,” Scientific Reports 11, no. 1 (2021): 22443, 10.1038/s41598-021-01986-w. 34789833 PMC8599852

[47] N. L. Madureira , “Reckless Proliferation and Guardianship Proliferation: The Fast Breeder Nuclear Reactor and the Plutonium Economy,” Technology and Culture 60, no. 3 (2019): 833–865, 10.1353/tech.2019.0075. 31422968

[48] Y. Zhang , C. Wang , Z. Lan , et al., “Review of Thermal‐Hydraulic Issues and Studies of Lead‐based Fast Reactors,” Renewable and Sustainable Energy Reviews 120 (2020): 109625, 10.1016/j.rser.2019.109625.

[49] D.‐L. Zhang , X. Li , D. Jiang , et al., “Fluoride‐salt‐cooled Advanced Reactor (FuSTAR),” Energy 290 (2023): 130048, 10.1016/j.energy.2023.130048.

[50] M. I. Ojovan , “Challenges in Long‐term Behavior of Radioactive Materials,” Sustainability 14, no. 4 (2022): 2445, 10.3390/su14042445.

[51] T. S. Melichar , P. Luksová , and M. Silhan , “A Proposal for Advanced Supplementary Technologies and a Hybrid System with Gas‐cooled Fast Reactor Concept ALLEGRO,” Nuclear Engineering and Design 415 (2023): 112645, 10.1016/j.nucengdes.2023.112645.

[52] J. Kupecki , J. Hercog , K. Motyliński , et al., “Hydrogen Production Using Nuclear Cycles and HTGR Integration,” International Journal of Hydrogen Energy 53 (2024): 40–48, 10.1016/j.ijhydene.2023.12.017.

[53] A. I. Arvanitidis , V. Agarwal , and M. Alamaniotis , “Nuclear‐Driven Integrated Energy Systems: A State‐of‐the‐Art Review,” Energies 16, no. 11 (2023): 4293, 10.3390/en16114293.

[54] G. C. Masotti , A. Cammi , S. Lorenzi , and M. E. Ricotti , “Modeling and Simulation of Nuclear Hybrid Energy Systems Architectures,” Energy Conversion and Management 298 (2023): 117684, 10.1016/j.enconman.2023.117684.

[55] R. S. Cherry , S. E. Aumeier , and R. D. Boardman , “Large Hybrid Energy Systems for Making Low CO_2_ Load‐Following Power and Synthetic Fuel,” Energy Environment Science 5, no. 2 (2012): 5489–5497, 10.1039/c1ee02731j.

[56] M. Koç , Y. E. Yuksel , and M. Ozturk , “Multigenerational Nuclear Energy Plant Analysis,” International Journal of Energy Research 46, no. 14 (2022): 20650–20669, 10.1002/er.7645.

[57] R. J. Taylor , W. Bodel , A. Banford , et al., “Sustainability of Nuclear Energy: UK Perspective,” Sustainability 16, no. 24 (2024): 10952, 10.3390/su162410952.

[58] A. A. Galahom , A. S. Khaliil , N. Alnassar , et al., “Thorium‐based Fuels for APR‐1400 Reactor,” Nuclear Engineering and Design 417 (2024): 112817, 10.1016/j.nucengdes.2023.112817.

[59] C. Wulandari , N. Trianti , S. Permana , and A. Waris , “Neutronic Design Performance of 100 MWe MSR with Thorium‐enriched Uranium and Thorium‐plutonium‐minor Actinide Fuel,” Nuclear Engineering and Design 414 (2023): 112646, 10.1016/j.nucengdes.2023.112646.

[60] B. S. McDonald , A. Danagoulian , A. J. Gilbert , et al., “Neutron Resonance Transmission Analysis Prototype System for Thorium Fuel Cycle Safeguards,” Nuclear Instruments and Methods in Physics Research Section A: Accelerators, Spectrometers, Detectors and Associated Equipment 1062 (2024): 169148, 10.1016/j.nima.2024.169148.

[61] E. Dewita , Suwoto , Zuhair , et al., “Implementation of Thorium‐Based Fuel for Indonesia Micro Reactor (IMR),” Nuclear Engineering and Design 425 (2024): 113334, 10.1016/j.nucengdes.2024.113334.

[62] S. U. Khan , Z. Almutairi , M. Alanazi , et al., “Techno‐economic Assessment of Modular Reactor Fuel Cycle,” Sustainability 13, no. 21 (2021): 11815, 10.3390/su132111815.

[63] I. N. Kessides and V. V. Kuznetsov , “SMRs for Energy Security in Developing Countries,” Sustainability 4, no. 8 (2012): 1–27, 10.3390/su4081806.

[64] S. Hong and B. W. Brook , “Economic Feasibility of Energy Supply by Small Modular Nuclear Reactors on Small Islands: Case Studies of Jeju, Tasmania and Tenerife,” Energies 11, no. 10 (2018): 2587, 10.3390/en11102587.

[65] W. R. Stewart and K. Shirvan , “Capital Cost Estimation for Advanced Nuclear Power Plants,” Renewable & Sustainable Energy Reviews 155 (2021): 111880, 10.1016/j.rser.2021.111880.

[66] G. A. Black , F. Aydogan , and C. L. Koerner , “Economic Viability of Light Water Small Modular Nuclear Reactors: General Methodology and Vendor Data,” Renewable and Sustainable Energy Reviews 103 (2019): 248–258, 10.1016/j.rser.2018.12.041.

[67] T. J. Lindroos , E. Pursiheimo , V. Sahlberg , and V. Tulkki , “A Techno‐Economic Assessment of NuScale and DHR‐400 Reactors in a District Heating and Cooling Grid,” Energy Sources, Part B: Economics, Planning, and Policy 14, no. 1 (2019): 13–24, 10.1080/15567249.2019.1595223.

[68] P. Fernández‐Arias , D. A. Vergara , Á. Antón‐Sancho , et al., “Bibliometric Review of PWR SMRs,” Energies 16, no. 13 (2023): 5168, 10.3390/en16135168.

[69] K. Kiegiel , I. Herdzik‐Koniecko , L. Fuks , and G. Zakrzewska‐Koltuniewicz , “Management of Radioactive Waste from HTGR Reactors Including Spent TRISO Fuel—State of the Art,” Energies 15, no. 3 (2022): 1099, 10.3390/en15031099.

[70] B. Merk and D. Litskevich , “A Disruptive Approach to Eliminating Weapon‐grade Plutonium – Pu Burning in a Molten Salt Fast Reactor,” PLOS One 13, no. 8 (2018): 0201757, 10.1371/journal.pone.0201757. PMC6072088 30071010

[71] W. H. Hannum , “Modern and Future Nuclear Fuel Cycles and the Relationship with Nuclear Waste Management,” WIREs Energy and Environment 3, no. 4 (2014): 323–329, 10.1002/wene.99.

[72] R. Taylor , W. Bodel , L. Stamford , and G. Butler , “A Review of Environmental and Economic Implications of Closing the Nuclear Fuel Cycle—Part One: Wastes and Environmental Impacts,” Energies 15, no. 4 (2022): 1433, 10.3390/en15041433.

[73] M. Oettingen , et al., “Radiotoxicity Assessment of Spent Nuclear Fuel,” Energies 14, no. 11 (2021): 3094, 10.3390/en14113094.

[74] M. Alyapyshev , V. Babain , and D. Kirsanov , “Isolation and Purification of Actinides Using N,O‐Hybrid Donor Ligands for Closing the Nuclear Fuel Cycle,” Energies 15 (2022): 7380, 10.3390/en15197380.

[75] M. A. Rahmanta , A. W. Harto , A. Agung , and M. K. Ridwan , “Nuclear Power Plant to Support Indonesia's Net Zero Emissions: A Case Study of Small Modular Reactor Technology Selection Using Technology Readiness Level and Levelized Cost of Electricity Comparing Method,” Energies 16, no. 9 (2023): 3752, 10.3390/en16093752.

[76] B. Poudel , K. Joshi , and R. Gokaraju , “A Dynamic Model of Small Modular Reactor Based Nuclear Plant for Power System Studies,” IEEE Transactions on Energy Conversion 35, no. 2 (2020): 977–985, 10.1109/tec.2019.2956707.

[77] M. K. Rowinski , T. J. White , and J. Zhao , “Small and Medium Sized Reactors (SMR): A Review of Technology,” Renewable and Sustainable Energy Reviews 44 (2015): 643–656, 10.1016/j.rser.2015.01.006.

[78] C. Zeliang , Y. Mi , A. Tokuhiro , L. Lu , and A. Rezvoi , “Integral PWR‐Type Small Modular Reactor Developmental Status, Design Characteristics and Passive Features: A Review,” Energies 13, no. 11 (2020): 2898, 10.3390/en13112898.

[79] A. Kadiyala , R. R. Kommalapati , and Z. Huque , “Lifecycle Greenhouse Gas Emissions of Nuclear Power Systems,” Energies 9, no. 11 (2016): 863, 10.3390/en9110863.

[80] E. S. Warner and G. A. Heath , “Life Cycle Greenhouse Gas Emissions of Nuclear Electricity Generation,” Journal of Industrial Ecology 16, no. 1 (2012), 10.1111/j.1530-9290.2012.00472.x.

[81] T. Gibon and Á. Hahn Menacho , “Parametric Life Cycle Assessment of Nuclear Power for Simplified Models,” Environmental Science & Technology 57 (2023): 14194–14205, 10.1021/acs.est.3c03190. 37698276 PMC10537461

[82] A. Moonesi Shabestary , F. Viereckl , Y. Zhang , et al., “Modeling Passive Heat Removal Systems,” Energies 13, no. 1 (2019): 35, 10.3390/en13010035.

[83] X. Zhang , M. Hu , Y. Zhang , et al., “Thermal‐Hydraulic Analysis of Passive Safety Features,” Nuclear Engineering and Design 416 (2024): 112764, 10.1016/j.nucengdes.2023.112764.

[84] F. Di Maio , N. Pedroni , B. Tóth , L. Burgazzi , and E. Zio , “Reliability Assessment of Passive Safety Systems,” Energies 14, no. 15 (2021): 4688, 10.3390/en14154688.

[85] S. Passerini and M. S. Kazimi , “Sustainability Features of Nuclear Fuel Cycles,” Sustainability 4, no. 10 (2012): 2377–2398, 10.3390/su4102377.

[86] J. Roth and J. d'Angelo , “Nuclear Materials and Energy Overview,” Nuclear Materials and Energy 6 (2016): 10–11, 10.1016/j.nme.2015.11.001.

[87] T. H. S. C. Afonso , G. G. G. Fontes , M. L. Moreira , and D. A. P. Palma , “Performance Evaluation of Nuclear Fuel in a Reactor Based on the CAREM 25,” Nuclear Engineering and Design 415 (2023): 112661, 10.1016/j.nucengdes.2023.112661.

[88] Z. Zhang , T. Wu , Y. Wang , S. Chen , C. Yuan , and J. Zhu , “Core Design and Neutronic Study on Small Reactor with Advanced Fuel Designs,” Nuclear Materials and Energy 29 (2021): 101068, 10.1016/j.nme.2021.101068.

[89] S. A. Hosseini , R. Akbari , A. S. Shirani , and F. D'Auria , “SMR Licensing Process Based on BEPU Approach,” Sustainability 15, no. 8 (2023): 6636, 10.3390/su15086636.

[90] B. Taebi , M. van Asselt , and I. van de Poel , “Multilateral Governance of Technological Risks; Editors′ Overview 1,” Journal of Risk Research 25, no. 8 (2022): 941–944, 10.1080/13669877.2022.2104345.

[91] J. R. Katzer , “Sustainable Research, Development, and Demonstration (RD&D),” Industrial & Engineering Chemistry Research 49, no. 21 (2010): 10154–10158, 10.1021/ie1005965.

[92] G. Federici , W. Biel , M. R. Gilbert , R. Kemp , N. Taylor , and R. Wenninger , “European DEMO Design Strategy and Consequences for Materials,” Nuclear Fusion 57, no. 9 (2017): 092002, 10.1088/1741-4326/57/9/092002.

[93] J.‐W. Wang , “Nuclear Power Technologies Development and Future Evolution,” Journal of Cleaner Production 439 (2024): 140915, 10.1016/j.jclepro.2024.140915.

[94] R. Petroski and L. Wood , “Sustainable Nuclear Fission Energy at Planetary Scale,” Sustainability 4, no. 11 (2012): 3088–3123, 10.3390/su4113088.

[95] G. Federici , C. Bachmann , L. Barucca , et al., “Overview of the DEMO Staged Design Approach in Europe,” Nuclear Fusion 59, no. 6 (2019): 066013, 10.1088/1741-4326/ab1178.

[96] K. Gupta , J. Ripberger , A. Fox , et al., “Risk Communication and Public Response to Radiation Emergencies,” Risk Analysis 45 (2024): 1237–1253, 10.1111/risa.17657. 39380450

[97] M. P. Mohan and S. K. Namboodhiry , “Public Perception of Nuclear Energy in India,” Journal of Public Affairs 20, no. 3 (2020): 2086, 10.1002/pa.2086.

[98] L. Zhou and Y. Guo , “Risk Communication Policy Design and Public Perception,” Journal of Comparative Policy Analysis 27 (2025): 1–20, 10.1080/13876988.2024.2442575.

[99] R. Bhattacharyya and F. Khalid , “Rapid Assessment of Integrated Nuclear Cogeneration Projects Using Multi‐Criteria Indices,” International Journal of Energy Research 45, no. 12 (2021): 17647–17663, 10.1002/er.7012.

[100] J. Buongiorno , M. Corradini , J. Parsons , and D. Petti , “Nuclear Energy in a Carbon‐Constrained World: Big Challenges and Big Opportunities,” IEEE Power and Energy Magazine 17, no. 2 (2019): 69–77, 10.1109/mpe.2018.2885250.

[101] D. Yoon , “Corrosion Behavior of Materials for Molten Salt Reactors,” International Journal of Energy Research 2024 (2024): 2883918, 10.1155/2024/2883918.

[102] F. P. Cusmanri and H. Lim , “Licensing Process for Floating Nuclear Power Plants,” Energies 17 (2024): 5628, 10.3390/en17225628.

[103] Q. Wang , X. Chen , and X. Yi‐Chong , “Accident Like the Fukushima Unlikely in a Country with Effective Nuclear Regulation: Literature Review and Proposed Guidelines,” Renewable and Sustainable Energy Reviews 17 (2013): 126–146, 10.1016/j.rser.2012.09.012.

[104] B. Mignacca and G. Locatelli , “Economics and Finance of Small Modular Reactors: A Systematic Review and Research Agenda,” Renewable and Sustainable Energy Reviews 118 (2020): 109519, 10.1016/j.rser.2019.109519.

[105] M. A. Winker , T. Bloom , S. Onie , and J. Tumwine , “Equity, Transparency, and Accountability: Open Science for the 21st Century,” The Lancet 402 (2023): 1206–1209, 10.1016/s0140-6736(23)01575-1. 37805196

[106] X. Nie , M. Cheng , X. Zuo , and Z. Dai , “Multi‐Objective Capacity Configuration Optimization of a Nuclear‐Renewable Hybrid System,” Applied Thermal Engineering 248 (2024): 123365, 10.1016/j.applthermaleng.2024.123365.

[107] B. Zakeri , S. Rinne , and S. Syri , “Wind Integration with Nuclear Power Systems,” Energies 8, no. 4 (2015): 2493–2527, 10.3390/en8042493.

[108] G. S. Frankel , J. D. Vienna , J. Lian , et al., “Recent Advances in Corrosion Science Applicable to Disposal of High‐Level Nuclear Waste,” Chemical Reviews 121, no. 20 (2021): 12327–12383, 10.1021/acs.chemrev.0c00990. 34259500

[109] Y. Xie , J. Wang , Y. Hu , et al., “Corrosion and Contamination of 316L Stainless Steel in Simulated HNO_3_‐Based Spent Nuclear Fuel Reprocessing Environments with Cesium and Strontium,” Industrial & Engineering Chemistry Research 61, no. 26 (2022): 9342–9355, 10.1021/acs.iecr.2c01424.

[110] T. Wenga , W. Gwenzi , I. A. Jamro , and W. Ma , “High‐Temperature Corrosion‐Resistant Alloys for Waste‐to‐Energy Plants,” Heliyon 10 (2024): e30177, 10.1016/j.heliyon.2024.e30177. 38707319 PMC11068605

